# NAIGO: An Improved Method to Align PPI Networks Based on Gene Ontology and Graphlets

**DOI:** 10.3389/fbioe.2020.00547

**Published:** 2020-06-19

**Authors:** Lijuan Zhu, Ju Zhang, Yi Zhang, Jidong Lang, Ju Xiang, Xiaogang Bai, Na Yan, Geng Tian, Huajun Zhang, Jialiang Yang

**Affiliations:** ^1^College of Mathematics and Computer Science, Zhejiang Normal University, Jinhua, China; ^2^Institute of Infectious Diseases, Beijing Ditan Hospital, Capital Medical University, and Beijing Key Laboratory of Emerging Infectious Diseases, Beijing, China; ^3^Department of Mathematics, Hebei University of Science & Technology, Shijiazhuang, China; ^4^Geneis Beijing Co., Ltd., Beijing, China; ^5^Neuroscience Research Center & Department of Basic Medical Sciences, Changsha Medical University, Changsha, China; ^6^School of Computer Science and Engineering, Central South University, Changsha, China

**Keywords:** PPI network, network alignment, gene ontology, graphlets, functional ortholog

## Abstract

With the development of high throughput technologies, there are more and more protein–protein interaction (PPI) networks available, which provide a need for efficient computational tools for network alignment. Network alignment is widely used to predict functions of certain proteins, identify conserved network modules, and study the evolutionary relationship across species or biological entities. However, network alignment is an NP-complete problem, and previous algorithms are usually slow or less accurate in aligning big networks like human vs. yeast. In this study, we proposed a fast yet accurate algorithm called Network Alignment by Integrating Biological Process (NAIGO). Specifically, we first divided the networks into subnets taking the advantage of known prior knowledge, such as gene ontology. For each subnet pair, we then developed a novel method to align them by considering both protein orthologous information and their local structural information. After that, we expanded the obtained local network alignments in a greedy manner. Taking the aligned pairs as seeds, we formulated the global network alignment problem as an assignment problem based on similarity matrix, which was solved by the Hungarian method. We applied NAIGO to align human and *Saccharomyces cerevisiae* S288c PPI network and compared the results with other popular methods like IsoRank, GRAAL, SANA, and NABEECO. As a result, our method outperformed the competitors by aligning more orthologous proteins or matched interactions. In addition, we found a few potential functional orthologous proteins such as RRM2B in human and DNA2 in *S. cerevisiae* S288c, which are related to DNA repair. We also identified a conserved subnet with six orthologous proteins EXO1, MSH3, MSH2, MLH1, MLH3, and MSH6, and six aligned interactions. All these proteins are associated with mismatch repair. Finally, we predicted a few proteins of *S. cerevisiae* S288c potentially involving in certain biological processes like autophagosome assembly.

## Introduction

With the development of high-throughput techniques, such as yeast two-hybrid system (Uetz et al., 2000; Ito et al., 2001) and coimmunoprecipitation coupled to mass spectrometry (Krogan et al., 2006), a large amount of protein–protein interaction (PPI) networks in many species (e.g., human, yeast, and mouse) have been reported. Their nodes vary from hundreds to tens of thousands. Within a PPI network, each node denotes a protein, and each edge denotes an interaction between the two proteins connected. Interspecies network alignment is important for predicting protein functions (Dutkowski and Tiuryn, 2007; Singh et al., 2008) and understanding the PPI network evolutions (Flannick et al., 2006; Kuchaiev and PrŽulj, 2011). Many network alignment tools have been proposed. According to their comparison range, they could be categorized into local alignment and global alignment. The goal of local alignment is to find subnets conserved in two or more species, which are usually limited to a relative small range and involve only proteins with specific functions (Berg and Lässig, 2004, 2006; Brian et al., 2004; Sharan et al., 2005; Liang et al., 2006; Ciriello et al., 2012; Mina and Guzzi, 2014). On the contrary, the global alignment aims to find mappings traversing all nodes (Klau, 2009; Liao et al., 2009; Zaslavskiy et al., 2009; Milenković et al., 2010; Patro and Kingsford, 2012; Neyshabur et al., 2013; Faisal et al., 2015).

Since PPI network alignment is usually a generalized subgraph isomorphism problem, that is NP-hard (Lathrop, 1994), developing heuristic algorithms with good practical efficiency has become one of the foremost challenges. The existing network alignment algorithms could be classified into three categories: heuristic search method based on graph model, constraint optimization method based on objective function, and modular method based on the divide and conquer strategy. The heuristic search methods could establish interspecies alignment graphs with the orthologous proteins as nodes. They evaluate the similarity between PPI networks and design heuristic search algorithms generally adopting the greedy strategy of seed growth. Following similar ideas, different alignment tools also use different analytic strategies and algorithms. For example, MaWISH (Koyuturk et al., 2006) converts the local alignment into a maximum weight induced subgraph problem. Græmlin (Flannick et al., 2006) determines the initial matched proteins based on the log-likelihood ratio probability model and then gradually searches other similar protein nodes to expand the alignment graphs. The optimization-based methods could convert the alignment problem into an optimization problem. For instance, IsoRank (Singh et al., 2008) calculates the PPI network similarity with an eigenvalue matrix and extracts the global alignment from it. MNAligner (Li et al., 2007) converts the network alignment into an integer quadratic programming problem, while GRAAL (Kuchaiev et al., 2010) converts it into a topological structure problem. The other alignment tool, BinAligner (Yang et al., 2013), constructs a similarity matrix based on node similarity and “*n*-neighborhood” (*n* ≥ 1) subnet similarity and implements alignment by integer programming. The modular-based methods are also widely used for alignments, considering the large size of PPI networks and their modular structures (Hartwell et al., 1999; Silva and Stumpf, 2005; Sharan and Ideker, 2006; Almaas, 2007; Srinivasan et al., 2007). For example, Match-and-Split alignment (Narayanan and Karp, 2007) divides the modules by matching and splitting, while BiNA (Towfic et al., 2009) divides the original network into multiple subnets and aligns them by a kernel function subsequently.

Although many algorithms for PPI network alignment have been developed, most current tools either have lower accuracy or fail to align large networks. There is still a need for a fast and accurate alignment algorithm. In this study, we propose an improved alignment method, NAIGO, which integrates divide-and-conquer strategy, optimization modeling, and graph-based features. It can align PPI networks locally and globally based on the node similarity, edge similarity, and topological similarity of the networks. To improve the calculation efficiency, NAIGO achieves the global alignment between large networks by expanding prealigned subnets in a greedy manner. In contrast to other alignment algorithms, NAIGO could also expand the smaller subnets by referring to the matched bigger ones and thus predict the unknown biological process (BP) of proteins. We applied NAIGO to align the PPI networks of human and *Saccharomyces cerevisiae* S288c. Compared with other popular methods such as GRAAL (Kuchaiev et al., 2010), IsoRank (Singh et al., 2008), SANA (Mamano and Hayes, 2017), and NABEECO (Ibragimov et al., 2003), NAIGO has a better alignment performance.

## Methods

Our algorithm consists of three steps: (1) divide the large networks into multiple small subnets, (2) align the corresponding subnets based on the similarity matrix, (3) expand the interspecies alignment graphs based on the heuristic search idea, with the best aligned subnets as nodes.

### Network Division

**Figure 1:**
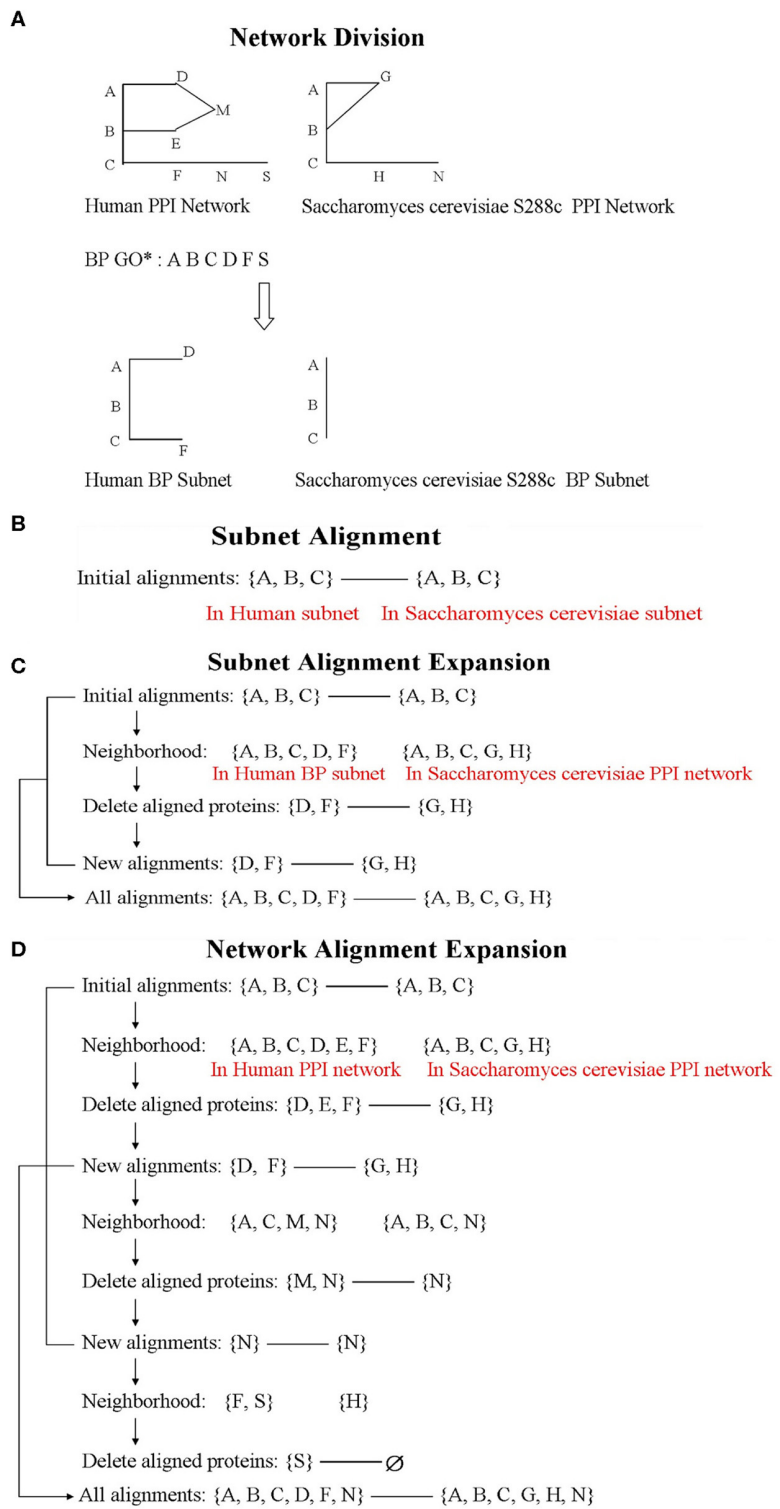
The flowchart of Network Alignment by Integrating Biological Process (NAIGO). The letters, such as **(A–C)**, represent proteins in the figure. **(D)** Network alignment expansion.

Considering that similar proteins participate in the same biological process in different species, we use the BP information of Gene Ontology (GO) as the criteria to divide the network. In our study, BP data was extracted by loading the GO.db package, and it contained a total of 14,291 GO terms. Based on the BP terms, we divided the network as follows: if two interacted proteins both involved in the same term, they will be included in the subnet (Figure 1A). The division method could avoid isolated vertices. According to the criteria, the PPI network of human could be divided into 6,781 non-empty subnets, and the PPI network of *S. cerevisiae* S288c could be divided into 1,836 non-empty subnets. Among them, there are 1,771 subnet pairs under the same terms of the two species. In the corresponding 1,771 terms, except for the biological process term, only one term pair had containment relationship, and the included term was removed. Thus, we consider aligning the 1,770 BP subnet pairs (i.e., align human subnet *i* to *S. cerevisiae* S288c subnet *i*, *i* = 1, 2, …, 1, 770) (Figure 1B).

### Subnet Alignment Based on Similarity Matrix

A PPI network is an undirected graph *G* = (*V, E*), where the node set *V* represents the proteins and the edge set *E* represents interactions between proteins. Given two PPI subnets, *G* = (*V, E*) and *H* = (*U, F*), the subnet alignment is defined as a one-to-one mapping (π) between node set *V* and *U*.

(1) $$\begin{array}{l} \pi :\left\{i\in V\right\}\mapsto \left\{j\in U\right\} \end{array}$$

where π reflects the alignment of proteins, and we define the mapping value as Equation (2). If we get the π_*ij*_ value of each protein pair in the two PPI subnets (*i* ∈ *V*, *j* ∈ *U*) and confirm the correspondence of each interaction pair based on π_*ij*_, we will achieve a subnet alignment.

(2) $$\begin{array}{l} \pi_{ij}=\{\begin{array}{c} 1,\;if\;j=\pi \left(i\right)\\ 0,\;otherwise \end{array} \end{array}$$

To get the optimal alignment efficiently, we constructed a similarity matrix (*C*) for each PPI subnet pair. We need to solve the integer linear programming problem to maximize the alignment score *S* in Equation (3), which is subject to restrictions as Equation (4). Hungarian algorithm was adopted to solve the problem.

(3) $$\begin{array}{l} \underset{\;\pi }{S=\max\;}\underset{i\in V}{\sum }\underset{j\in U}{\sum }C_{ij}\pi_{ij} \end{array}$$

Subject to

(4) $$\begin{array}{l} \{\begin{array}{l} \sum_{i\in V}\pi_{ij}=0\;\text{or }1,\;\forall j\in U\\ \sum_{j\in U}\pi_{ij}=0\;\text{or }1,\;\forall i\in V\\ \pi_{ij}=0\;\text{or }1,\;\forall i\in V\;\text{and }j\in U \end{array} \end{array}$$

In the above equation, *V* and *U* represent the node set of subnet *G* and *H*, respectively.

### Construction of Similarity Matrix

Obviously, the PPI subnet alignments were decided by their topological structure, as well as the conservation of the proteins (nodes) and interactions (edges) that mainly influenced by the protein orthology. Therefore, the similarity matrix *C* considers both the protein orthology and network topology as follows.

(5) $$\begin{array}{l} C=\theta_{1}*\left(A+A^{*}\right)+\theta_{2}*B\left(0\leq \theta_{1},\theta_{2}\leq 1\;\text{and }\theta_{1}+\theta_{2}=1\right) \end{array}$$

On the one hand, we define the corresponding protein orthology matrix as *A* and satisfies Equation (6). The orthologous file between *S. cerevisiae* S288c and human was downloaded from Ensembl (http://www.ensembl.org/biomart/martview). Besides, we also lead A^*^ into the similarity matrix and define it as Equation (7), which reflects the contribution of the orthologous protein pairs to the edge matching. Within it, *N*(*i*) is the set of neighbors of node *i*, |*N*(*i*)| is the size of this set, and *N*(*i*) × *N*(*j*) is the Cartesian product of sets *N*(*i*) and *N*(*j*).

(6) $$\begin{array}{lll} A\left(i,j\right) & = & \{\begin{array}{l} 1\;\text{if }i\;and\;j\;\text{are orthologs}\\ 0\;otherwise \end{array} \end{array}$$

(7) $$\begin{array}{lll} A^{*}\left(i,j\right) & = & \frac{sum\left\{A\left[N\left(i\right)\;\times N\left(j\right)\right]\right\}}{\left|N\left(i\right)|*|N\left(j\right)\right|} \end{array}$$

On the other hand, we also compare the topological structure around the nodes during the subnet alignment, which is based on the graphlet degree (PrŽulj, 2007). A graphlet is a connected non-isomorphic subgraph of a large network, in which non-automorphism positions are called orbits. We construct graphlet matrix *G*_*n* × 73_ based on 30 (2-, 3-, 4-, and 5-node) graphlets with 73 orbits, where *n* is the number of nodes in the subnet and *G*(*i, j*) is the number of graphlets touched by node *i* at orbit *j*−1 (*j* = 1, 2, ..., 73). Furthermore, the graphlet similarity matrix is defined as *B* and satisfies

(8) $$\begin{array}{l} B\left(i,j\right)=\frac{1}{1+\sqrt{\overset{73}{\underset{k=1}{\sum }}{\left[G_{1}\left(i,k\right)-G_{2}\left(j,k\right)\right]}^{2}}} \end{array}$$

*G*_1_ and *G*_2_ represent the graphlet matrices of two species. The number of rows is the number of proteins in the subnet, and the number of columns is the number of orbits.

### Parameter Setting of Similarity Matrices

As displayed in Equation (5), how to weight the protein orthology (θ_1_) and network topology (θ_2_) is essential for constructing the similarity matrices. We consider the protein orthology as the major determinant when there are many orthologous protein pairs between corresponding subnets. Otherwise, we consider the topology as the major determinant. We thus calculate the weighting coefficient as follows (let *m* be the number of subnets).

(9) $$\begin{array}{lll} \theta_{1} & = & \{\begin{array}{ll} p_{i} & \text{if }p_{i}<1,\\ \underset{j=1,2,...,m}{\text{mean}}\left(p_{j}|p_{j}<1\right) & \text{else}.\; \end{array} \end{array}$$

(10) $$\begin{array}{lll} \theta_{2} & = & 1-\theta_{1} \end{array}$$

where *p* represents the orthologous protein ratio and is calculated with Equation (11), where *n*, *n*_1_, and *n*_2_ represents the number of orthologous protein pairs and the number of proteins in two PPI subnets, respectively.

(11) $$\begin{array}{l} p=\frac{n}{\min\left(n_{1},n_{2}\right)} \end{array}$$

When *p*_*i*_ < 1 in the subnet, the weighting coefficient of the protein orthology (θ_1_) is equal to *p*_*i*_; otherwise, θ_1_ is the mean value of *p*_*i*_ of the other subnets with *p*_*i*_ < 1.

### Simplification of Similarity Matrices

When the dimension of matrix *C* is large, we simplify it to improve the calculation speed without affecting results. Let the matrix dimension be *m* × *n*. If *m* = *n*, *C* could not be simplified. Otherwise, the simplification principles are as follows, taking *m*>*n* as an example.

(1) Extract the rows with orthologous proteins from *C*. Let the number of rows be *r*_1_. The extracted matrix and remaining matrix are denoted by *Z*_1_ and *C*_1_, respectively.

(2) Extract the rows from *C*_1_ which include the largest element of each column. Let the number of rows be *r*_2_. The extracted matrix and remaining matrix are denoted by *Z*_2_ and *C*_2_, respectively.

(3) If *r*_1_ + *r*_2_ ≥ *n*, [*Z*_1_; *Z*_2_] is the simplified matrix *C*. If *r*_1_ + *r*_2_ < *n*, extract the rows from *C*_2_ according to the step (2). Repeat the matrix extraction and test until $\sum_{q=1}^{Q}r_{q}\geq n$.

### The Whole Network Alignment Expansion

After the subnet alignments, we treat the largest aligned subnet as a seed and expand it with the heuristic search method until aligning all the nodes. Then, we could achieve the global alignment between large PPI networks. The expansion steps are as follows (Figure 1D):

(1) Let the seed of Human be *V* and its neighbor (i.e., the node set that interacts with *V*) be *V*_1_; the seed of *S. cerevisiae* S288c be *U* and its neighbor be *U*_1_. Remove the repeatedly occurring proteins in their corresponding seed from *V*_1_/*U*_1_ (due to the interactions between the proteins in *V*/*U*, there are overlapping proteins in *V*/*U* and *V*_1_/*U*_1_).
(2) Find the alignment between *V*_1_ and *U*_1_ by resolving the optimization problem and maximizing the alignment score *S* as Equation (12), and thus expand the aligned seed.
(3) Let the neighbor of *V*_1_ be *V*_2_ and the neighbor of *U*_1_ be *U*_2_. Repeat steps (1) and (2) until the seed network expands to *n* layers and *U*_*n*+1_ or *V*_*n*+1_ is empty. Merge all results to achieve the global alignment between two species.

The alignment score *S* is also the criterion for assessing alignment. However, we use the scoring scheme of graph alignment (Berg and Lässig, 2006; Kolár et al., 2008) in the whole network alignment expansion, comparing to the similarity matrix based strategy in the subnet alignment. It is achievable when the subnet alignment is implemented as a seed and is more accurate. Briefly, we define *S* as the sum of the nodes score *S*_*V*_ and edge score *S*_*E*_. In order to maximize *S*, we resolve the optimization problem with the Hungarian algorithm in Equation (12), which is subject to restrictions as Equation (13). Taking π_*ij*_ as the aligned seed, we could obtain the π_*kl*_ value for any *k* in *V* and *l* in *U* by resolving assignment problem.

(12) $$\begin{array}{l} S=\max\;\underset{k\in V}{\sum }\underset{l\in U}{\sum }\alpha_{kl}\pi_{kl}+\underset{i,k\in V}{\sum }\underset{j,l\in U}{\sum }\beta_{ijkl}\pi_{ij}\pi_{kl} \end{array}$$

Subject to

(13) $$\begin{array}{l} \{\begin{array}{l} \underset{i\in V}{\sum }\underset{j\in U}{\sum }C_{ij}\pi_{ij}=\overset{\hat{\;}}{s,}\\ \sum_{k\in V}\pi_{kl}=0\;\text{or }1,\;\forall l\in U\\ \sum_{l\in U}\pi_{kl}=0\;\text{or }1,\;\forall k\in V\\ \pi_{kl}=0\;\text{or }1,\;\forall k\in V\;\text{and }l\in U \end{array} \end{array}$$

In the above equation, *V* and *U* represent the node set of human and *S. cerevisiae* S288c PPI network, respectively. *S*_*V*_ scores protein pairs, in which the sequence similarity score (α_*kl*_) is used to determine the most likely orthologous proteins [*l* = π(*k*)]. *S*_*E*_ scores PPI pairs, in which β_*ijkl*_ is used to reward each matched PPI pair and punish each mismatched PPI pair. Given the nodes *i, k* ∈ *V* and *j, l* ∈ *U*, as well as the PPIs *ik* ∈ *E* and *jl* ∈ *F*, if *j* = π(*i*) and *l* = π(*k*), the PPIs *ik* and *jl* are matched edges; otherwise, they are mismatched edges. The parameter values are settled by Equations (14, 15), according to the reference (Yang et al., 2013).

(14) $$\begin{array}{lll} \alpha_{kl} & = & \{\begin{array}{l} 4.4\;k\;\text{and }l\;\text{are orthologs}\\ -1.6\;\text{else} \end{array} \end{array}$$

(15) $$\begin{array}{lll} \beta_{ijkl} & = & \{\begin{array}{l} 1.6\;\text{if }ik\in E\;\text{and }jl\in F\\ -0.3\;\text{else} \end{array} \end{array}$$

### The Subnet Alignment Expansion

The biological information of human PPI network is more complete, and the number of proteins in each subnet is greater than that of *S. cerevisiae* S288c. Therefore, the potential functions of many *S. cerevisiae* proteins could be further annotated by referring to the human subnets and only expanding the corresponding *S. cerevisiae* ones. The expansion steps are as follows (similar to the whole network expansion) (Figure 1C):

(1) Let the aligned protein set of *S. cerevisiae* subnet be *X* and its neighbor in whole PPI network be *X*_1_. If *X*_1_ contains the proteins of *X*, remove them from *X*_1_.
(2) Let the aligned protein set of human subnet be *Y* and its neighbor in BP subnet be *Y*_1_. Compare *X*_1_ and *Y*_1_ adopting the same solution method as the whole network expansion.
(3) According to the alignment results, the new aligned proteins of *X*_1_ are predicted to participate in the corresponding BP.

### Performance Assessment of Network Alignment

Node coverage (NC) has been widely used (Milenković et al., 2010) to evaluate how well an alignment reconstructs the true node mapping, and it is defined as the percentage of aligned orthologs in the node set of the smaller network (*U*). In addition, global network aligners often fail to align all the nodes of the smaller network to the larger one. Thus, we also use global node coverage (GNC) to evaluate global alignments, which measures the number of mapped nodes normalized by the number of nodes in the smaller network (Malod-Dognin et al., 2017). To measure how well edges are conserved under an alignment, three measures have been used to date: edge correctness (EC) (Kuchaiev et al., 2010), induced conserved structure (ICS) (Patro and Kingsford, 2012), and symmetric substructure score (*S*^3^) (Saraph and Milenković, 2014). *S*^3^ has been shown to be superior to EC and ICS, since, intuitively, not only it penalizes alignments from sparse graph regions to dense graph regions (as EC does) but also it penalizes alignments from dense graph regions to sparse graph regions (as ICS does). Hence, we only focus on *S*^3^ ($S^{3}=\frac{\left|E_{1}^{*}\right|}{\left|E_{1}\right|+\left|E_{2}^{\prime }\right|-\left|E_{1}^{*}\right|}$, where $\left|E_{1}^{*}\right|$ is the number of edges from smaller network *G*_1_ that are conserved by alignment, $\left|E_{2}^{\prime }\right|$ is the number of edges from the induced subnet of *G*_2_ with aligned node set). Besides, the alignment score *S* is also the criterion for assessing alignment comprehensively. We use GO correctness (GC) to evaluate the biological quality of an alignment. GC is defined as the percentage of aligned protein pairs that share at least *k* GO terms (*k* = 1, 2, …) (Kuchaiev et al., 2010). In our study, we choose *k* = 1. Since the subnet pairs are divided by the BP terms, the GCs of all subnet alignments are 1.

In addition, we also assess the alignment performance by exploring the functions of aligned proteins. The aligned proteins with similar functions will get higher evaluation. The other indicator for alignment quality is the number of found common subnets representing the conserved functional clusters.

## Results

### NAIGO Algorithm and Benchmark Datasets

The NAIGO algorithm has integrated divide-and-conquer strategy, optimization modeling, and graph-based features. When aligning large networks, it first divides them into multiple subnets based on the BPs of proteins and then locally aligns the corresponding subnets by constructing similarity matrices. The alignment problem is thus formulated as an assignment problem and could be solved by a polynomial time algorithm called the Hungarian method. The similarity matrices integrate the orthologous information and topological similarity information of the networks. It is worth mentioning that we added the edge matching information to the orthologous information. After all the subnet comparisons, we consider the largest interspecies aligned subgraphs as seeds and use the scoring scheme of graph alignment to expand them in a greedy manner. Then, we finally achieve the global alignment of large networks.

To test the NAIGO algorithm, human and *S. cerevisiae* S288c, two species separated by a long evolutionary distance, were picked out to perform the PPI network alignment. Although their PPIs have been widely explored, there is still a lack of studies on the PPI alignment between them, whether local or global. We downloaded the *S. cerevisiae* S288c data (BIOGRID-ORGANISM-Saccharomyces_cerevisiae_S288c-3.4.137.tab2.txt, see Dataset S1), human data (HPRD_PROTEIN_ INTERACTIONS_Build9.txt, see Dataset S1), and the orthologous file between these two species from BioGRID (Andrew et al., 2015), Human Protein Reference Database (HPRD) (Keshava et al., 2009), and Ensembl (http://www.ensembl.org/biomart/martview), respectively.

The human PPI network consisted of 9,465 proteins and 39,240 interactions, among which there were a total of 2,201 self-interactions and repeated interactions. We denoted it by a graph *G* = (*V, E*), in which |*V*|=9,465 and |*E*|=37,039 after removing the self-interactions and repeated interactions. In contrast, the *S. cerevisiae* S288c network consisted of 6,470 proteins and 342,123 interactions. We denoted it by *H* = (*U, F*), in which |*U*|=6,470 and |*F*|=228,703. According to the BPs of proteins, the human and *S. cerevisiae* S288c network were divided into 6,781 and 1,836 non-empty subnets, respectively. Among them, there were 1,770 overlapping subnets in the two species after removing the included subnet.

According to the orthologous file, there were 3,871 ortholog pairs between *V* and *U*. Since many proteins have more than one ortholog in the other species, we finally confirmed 1,908 non-overlapping ortholog pairs by Hungarian algorithm. Each protein was involved in up to one non-overlapping ortholog pair, which guaranteed accurate and efficient alignments.

### Effectiveness of Modified Algorithm and Defined Parameters

In NAIGO, the node and edge matching information were both introduced into the calculation of orthology (sequence-edge similarity), which was formulated into the similarity matrix together with the topological similarity. The parameters θ_1_ and θ_2_ were defined to balance contributions of nodes and edges.

This design ensured aligning more orthologous proteins on the matched edges and thus achieving better results. To evaluate the effectiveness of the integrative strategy and parameter setting, we randomly selected 30 subnet pairs. The interaction numbers of the smaller subnets ranged from 300 to 500. Adopting two common alignment scoring schemes, NC and *S*^3^, we tested the performances of (1) setting one parameter to one and the other to zero (i.e., θ_1_ = 0 and θ_2_ = 1), (2) setting the continuously changing parameters (0 < θ_1_ < 1 and θ_2_ = 1−θ_1_), and (3) defining parameters. The larger NC and *S*^3^, the better the performance. As shown in Table 1, both the sequence-edge and topological similarities contributed to the node and edge alignments, and the former played more important roles in it. Meanwhile, the defining parameters got the better NC and *S*^3^ performance. (For the defining parameters of 30 subnet pairs, see Table S1). The results demonstrated that the algorithm modification based on the sequence-edge similarity and defined parameters led to the good subnet alignment.

**Table 1:** The influence of balancing parameters on the alignment.

**θ_1_**	**θ_2_**	**Average NC**	**Average *S*^3^**
0	1	0.01	0.009
0.05	0.95	0.73	0.117
0.1	0.9	0.74	0.119
0.2	0.8	0.74	0.120
0.5	0.5	0.75	0.122
0.9	0.1	0.75	0.122
0.95	0.05	0.75	0.122
0.96	0.04	0.75	0.121
0.999	0.001	0.75	0.121
1	0	0.75	0.120
Defined θ_1_	Defined θ_2_	0.75	0.121

### Performance Assessment of Local Alignment

We adopted the shared 1,770 BP subnets in the human and *S. cerevisiae* to evaluate the local alignment performance of the NAIGO algorithm. The mean value (±SD) of the NC is 0.57 (±0.35), and the mean value (±SD) of the *S*^3^ is 0.30 (±0.33), the 1770 aligned subnets see Dataset S2 - aligned subnets.

**Figure 2:**
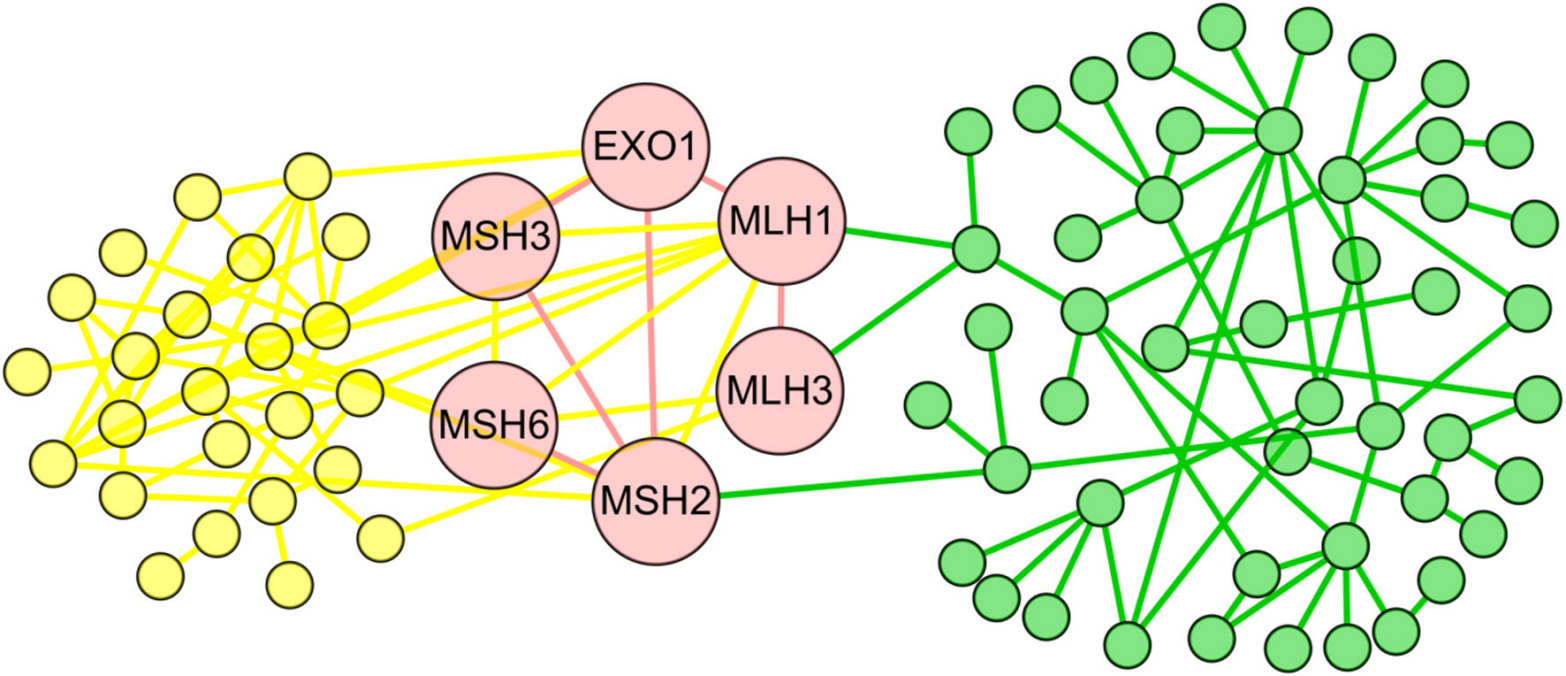
The conserved subnet. The yellow nodes and edges represent the proteins and interactions only existing in the *Saccharomyces cerevisiae* S288c, and the green nodes and edges represent the proteins and interactions only existing in human. The red nodes and edges form the conserved subnet of the two species.

**Figure 3:**
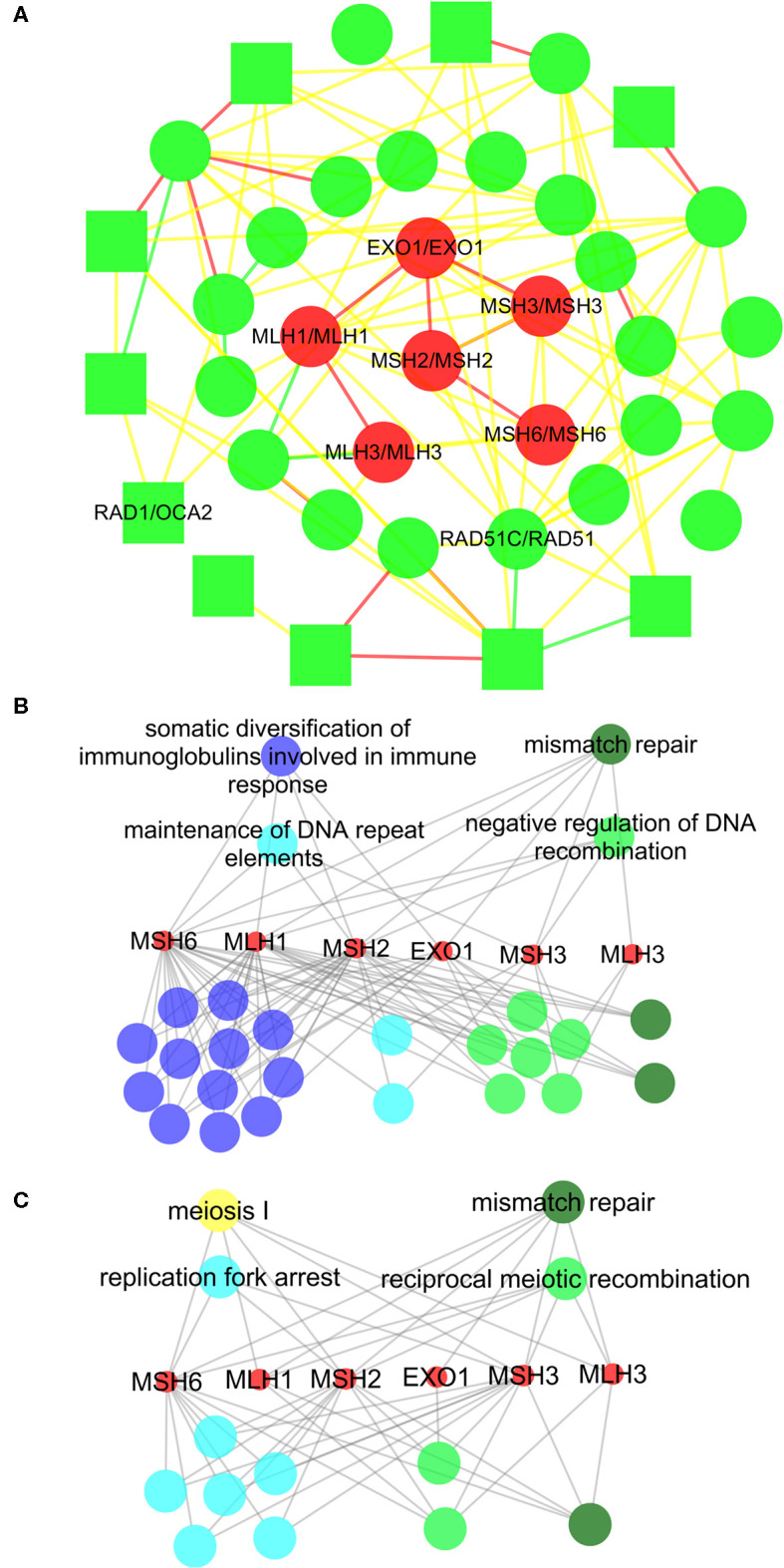
The subnet alignment and the functional annotation of the six orthologous proteins in the conserved subnets. In the subnet alignment **(A)**, the round and square nodes represent the orthologous and non-orthologous protein pairs, respectively. If the human protein “A” aligned to the Saccharomyces cerevisiae S288c protein “B,” then we represent the protein pair as “A/B.” The yellow edges represent the interactions only existing in the Saccharomyces cerevisiae S288c, and the green edges represent those only existing in human. The red edges are the matched interactions. We colored the nodes in red based on broad GO term mismatch repair that had the most proteins. ClueGO is used to perform the gene enrichment analysis based on biological process (BP). The annotated BP groups and terms in the human **(B)** and Saccharomyces cerevisiae S288c **(C)**, with the statistical significance (P < 0.05), are displayed separately. Each node represents a BP term, and each color represents a BP group. The dark green group represents mismatch repair; the light blue group represents DNA replication; the light green group represents DNA recombination; the dark blue group represents B-cell-mediated immunity; and the yellow group represents meiosis. For simplicity, we only remark the most significant BP term of each group in the figures. The edges indicate the involved proteins in each functional item.

Within them, we achieved the conserved subnets in 1,240 aligned subnet pairs, which included at least two aligned edge pairs. We randomly selected several conserved subnets as examples (Figure 2, Figures S1, S2, and Table S2). As shown in Figure 2, the conserved subnet contained six orthologous proteins (EXO1, MSH3, MSH2, MLH1, MLH3, and MSH6) and six aligned interactions (EXO1-MSH3, EXO1-MSH2, EXO1-MLH1, MSH3-MSH2, MLH3-MLH1, and MSH6-MSH2). Refer to Figure 3A for the alignment between the subnets where the conserved subnets are located, and we know that both the human and *S. cerevisiae* S288c proteins in conserved subnets are related to mismatch repair.

We annotated the functions of the six orthologous proteins in the human and *S. cerevisiae* S288c (Figures 3B,C), displaying the similar patterns in the two species. Using ClueGO, a Cytoscape plugin, the orthologous proteins were annotated into 26 and 13 BP terms in the human and *S. cerevisiae*, respectively. Within them, we found that all the six orthologous proteins involved in the Mismatch repair in the two species. Furthermore, the BP terms could be classified into five clusters, including three consistent ones, such as mismatch repair (the enrichment *p*-value is 2.52E−16 in human and 5.44E−15 in *S. cerevisiae*, respectively), DNA replication (7.09E−10/1.21E−08), and DNA recombination (1.32E−09/1.19E−09). It should be noted that the biggest BP cluster in the human, B-cell-mediated immunity, was not identified in the *S. cerevisiae* S288c. However, it was due to the corresponding BP terms mainly correlating to the somatic diversification of immune receptors/immunoglobulins via recombination and mutation, which had evolved only in vertebrates. We also performed Kyoto Encyclopedia of Genes and Genomes (KEGG) enrichment analysis on the six orthologous proteins in the human and *S. cerevisiae* S288c (Figure S3), and the results also showed that they were enriched in mismatch repair pathway. We also performed KEGG enrichment analysis on conserved subnets in Figures S1, S2 (see Figures S4, S5), and both subnet pairs had the same enrichment pathways. Therefore, these results indicated that the conserved subnets were functional conserved modules, and the NAIGO algorithm performed a correct local alignment.

In addition, the high performance of the NAIGO algorithm in the local alignment was also validated by annotating the functions of the non-orthologous protein pairs in the aligned subnets. We randomly selected five local alignments including 19 non-orthologous protein pairs and identified their functions by their BP terms and molecular function information on GeneCards. We totally found 10 non-orthologous protein pairs with similar functions (Table 2), indicating that these proteins were correctly aligned and should play similar roles in the two species.

**Table 2:** The functions of non-orthologous pairs.

**Human/*Saccharomyces cerevisiae* proteins**	**BP terms**	**GeneCards functions**
RRM2B/DNA2	DNA repair	Related to hereditary external ophthalmoplegia
LIG3/PIF1	NULL	DNA strand-break prevention and correction
RAD54L/CCT8	NULL	Telomere maintenance
RAD54B/REC114	Reciprocal meiotic recombination	Meiosis
AURKA/REC8	NULL	Structural maintenance of chromosome/spindle
RAD51/SPO11	Reciprocal meiotic recombination	NULL
TF/SCO1	NULL	Transportation
ATG16L1/ATG14	Protein transport Macroautophagy	Autophagy
GABARAP/ATG13	Autophagy Autophagosome assembly Macroautophagy	Neurotransmission
ARG2/CPS1	NULL	Urea metabolism

### Performance Assessment of Global Alignment

We also evaluate the NAIGO performance in the global alignment of the human and *S. cerevisiae* PPI networks. A total of 6,440 protein pairs have been aligned with the NAIGO algorithm, including the orthologous and non-orthologous protein pairs. It accounted for 68.04 and 99.54% of all proteins in the PPI networks of human and *S. cerevisiae* S288c, respectively. The NC of the global alignment is 0.21, the *S*^3^ is 0.02, and the GC is 0.15 (Table 3), the aligned network see Dataset S2 - aligned network.

**Table 3:** The predicted protein–protein interactions (PPIs) between subgraphs.

**Predicted human interactions**	**Corresponding *Saccharomyces* interactions**	**Alignment type**	**Experimental evidence**
VPS52-TSSK3	VPS52-AFT1	O-NO	*Science* paper (Menche et al., 2015)
VPS52-EPM2AIP1	VPS52-ERV41	O-NO	*Science* paper (Menche et al., 2015)
VPS52-COG3	VPS52-COG3	O-O	STRING database
VPS52-RAB6A	VPS52-YPT6	O-O	STRING database
VPS52-STX16	VPS52-TLG2	O-O	STRING database
VPS53-STX16	VPS53-TLG2	O-O	STRING database
VPS53-RAB6A	VPS53-YPT6	O-O	STRING database
USE1-STX5	USE1-SED5	O-O	STRING database
USE1-SCFD1	USE1-SLY1	O-O	STRING database
UBP1-ARHGAP21	UBP1-BEM2	O-O	STRING database

*O-O represents the PPI alignment with orthologous protein pairs as both nodes. O-NO represents the PPI alignment with an orthologous protein pair as one node and a non-orthologous protein pair as the other node*.

In addition, the NAIGO algorithm connected several fragmented subgraphs of the human PPI network by the global alignment. Within the HPRD database, the human PPI network consisted of 110 fragmented subgraphs, each of which had at least one edge, and had no interaction with each other. Correspondingly, there were two fragmented subgraphs in the PPI network of the *S. cerevisiae* S288c, one of which only had three vertices and three edges. After the global alignment, the aligned PPI graph covered eight and one fragmented subgraphs of the human and *S. cerevisiae*, respectively. On the one hand, we deduced 10 PPIs among the eight human fragmented subgraphs by comparing with the aligned *S. cerevisiae* network. Interestingly, we found the experimental evidences for all the 10 predicted interactions by retrieving them in the String database (https://string-db.org/) and the PPI network data compiled by Menche et al. (2015), which contained much more PPIs (>140,000) than the HPRD database we used. The predicted interactions are listed in the Table 3, indicating that the PPI network alignment by NAIGO could correctly deduced unknown PPIs. On the other hand, the unmatched fragmented subgraphs in the human PPI network were small, with nodes less than five and edges less than four. As most of the proteins of the *S. cerevisiae* S288c had been aligned to the human ones, we deduced that those unmatched human subgraphs correlated with the multicellular organism associated functions that had not evolved in *S. cerevisiae* yet. Taken together, we concluded that NAIGO could achieve the global alignment with high quality and predict some unknown PPIs accordingly.

### Comparison With Other Algorithms

To further assess the performance of the NAIGO algorithm, we compared it with four popular network alignment algorithms, IsoRank (Singh et al., 2008), GRAAL (Sharan et al., 2005), SANA (Mamano and Hayes, 2017), and NABEECO (Ibragimov et al., 2003). Within them, IsoRank calculates the network similarity by eigenvalue matrix and extracts the alignment, and GRAAL implements alignment only based on network topology structure. Both of the algorithms have a parameter to balance the contributions of the nodes and edges. Overall, NAIGO performed much better than the two algorithms. For IsoRank, we chose its default parameter to align the PPI networks of the human and *S. cerevisiae* S288c globally. As shown in Table 4, NAIGO achieved the larger NC/GNC, *S*^3^/*S* and GC than IsoRank. For GRAAL, we first randomly selected 30 subnet pairs, since it is hard for GRAAL to align the large networks. These pairs were the same with those for assessing the effectiveness of the NAIGO algorithm, with the interaction numbers of the smaller subnets ranging from 300 to 500. The GRAAL's parameter was optimized in steps of 0.1 from zero to one, and the best performance is listed in Table 4. Comparing with NAIGO, GRAAL's average NC performance of the 30 subnet pairs was significantly inferior, but its average *S*^3^ performance was better. Then, we compared the performances of the two methods only based on topology information (i.e., θ_1_ = 0 and θ_2_ = 1). The average NC of NAIGO was 0.01, which was better than that of GRAAL (0.002), but its average *S*^3^ (0.009) was worse than GRAAL's (0.15). The results reflected the misses of many orthologous protein pairs due to the better topology performance of GRAAL algorithm, which should be more obvious in the network alignments with large size differences. To balance the NC and *S*^3^ performances of GRAAL and thus compare with NAIGO better, we chose the other 15 subnet pairs. The protein numbers of the smaller subnets were >11,110, and the protein number differences of the protein pairs were <5. We also optimized the GRAAL's parameter in steps of 0.1 and found that GRAAL achieved much better NC performance without affecting the mean value of *S*^3^ (Table 4). Nevertheless, the NAIGO's NC and *S*^3^ performance in the alignment of these 15 subnet pairs were significantly better than GRAAL (Table 4). The performances of the two algorithms in global alignment and two sets of local alignment suggest that NAIGO performed substantially better than GRAAL.

**Table 4:** Comparison of five methods on aligning the PPI networks of human and *Saccharomyces cerevisiae* S288c.

**Method (the whole network pair)**	**NC/GNC**	***S*^3^/S**	**GC**
IsoRank	0.19/0.762	0.004/−2.6393E + 09	0.13
SANA	0/1	0.07/−2.6392E + 09	0.14
NAIGO	0.21/0.995	0.02/−2.6392E + 09	0.15
**Method (the first set of subnet pairs)**			
GRAAL	0.002	0.15	1
NABEECO	0.36	0.17	1
NAIGO	0.75	0.12	1
**Method (the second set of subnet pairs)**			
GRAAL	0.05	0.22	1
NAIGO	0.64	0.24	1

SANA and NABEECO are recent algorithms. Among them, SANA is a Simulated Annealing Network Aligner that can produce the alignment in a short time. NABEECO is a novel and robust Network Alignment heuristic based on Bee Colony Optimization. For SANA, we align the PPI networks of the human and *S. cerevisiae* S288c globally. SANA's GNC and *S*^3^ performance were better, but its NC and GC were inferior, especially that it missed all orthologous protein pairs. In addition, its largest connected component of aligned subgraph contained 6,118 nodes, while NAIGO contained 6,440 nodes, which indicated that the alignment result of NAIGO had stronger connectivity. For NABEECO, we choose the 30 subnet pairs to compare the performances because it needs to calculate the graphlet signature vector for long time when the network is large. Comparing with NAIGO, NABEECO's average NC performance of the 30 subnet pairs was significantly inferior, but its average *S*^3^ performance was better. It reflected that NABEECO resulted in high topology but low functional quality.

We compare the computational time of different methods based on the PPI networks of the human and *S. cerevisiae* S288c. The comparison results are shown in Table 5.

**Table 5:** The computational time of different methods.

**Method**	**SANA**	**IsoRank**	**NABEECO**	**GRAAL**	**NAIGO**
Time (h)	0.05	1.5	>72	>72	30

### Protein Function Deduction by Expanding Local Alignments

Due to the BP-based subnets, NAIGO could deduce the functions of certain proteins by expanding the local alignments. It was the unique advantage of the NAIGO algorithm, which could not be achieved by most of the existing alignment tools.

For a subnet of *S. cerevisiae* S288c, we predict that its aligned neighbor proteins will participate in the corresponding BP; thus, BP and subnet expansion was achieved. In order to evaluate the performances of BP and subnet expansion, we randomly selected 100 BPs and performed David analysis on the original BPs and the corresponding expanded BPs, of which 13 BPs were more significant, and 10 BPs had lower *P*-values. This shows that the BP proteins of *S. cerevisiae* S288c that we predicted are of practical significance.

**Figure 4:**
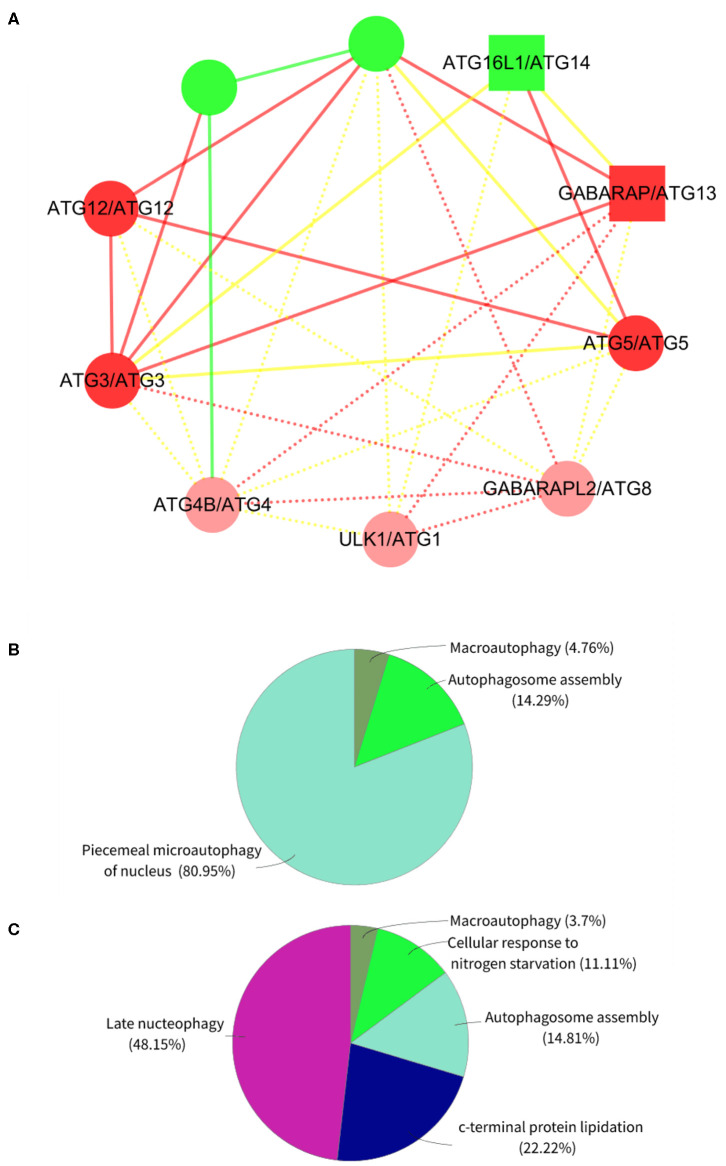
The expanded subnet alignment and the term group analysis of subnet in the *Saccharomyces cerevisiae* S288c. In the expanded subnet alignment **(A)**, the round and square nodes represent the orthologous and non-orthologous protein pairs, respectively. If the human protein “A” aligned to the *Saccharomyces cerevisiae* S288c protein “B,” then we represent the protein pair as “A/B.” The yellow edges represent the interactions only existing in the *Saccharomyces cerevisiae* S288c, and the green edges represent those only existing in human. The red edges are the matched interactions. We colored the nodes in red based on broad GO term autophagosome assembly that had most proteins. In addition, we represent the expanded proteins and interactions in *Saccharomyces cerevisiae* as light red nodes and dotted lines, respectively. ClueGO is used to perform the gene enrichment analysis based on biological process (BP). Each annotated BP group and its corresponding term percentage (the term percentage of a group is the ratio of the number of terms in the group to the number of terms in all groups) of subnet **(B)** and expanded subnet **(C)** are displayed. Each color represents a group, and we remark the most significant BP term of each group in the figures.

**Figure 5:**
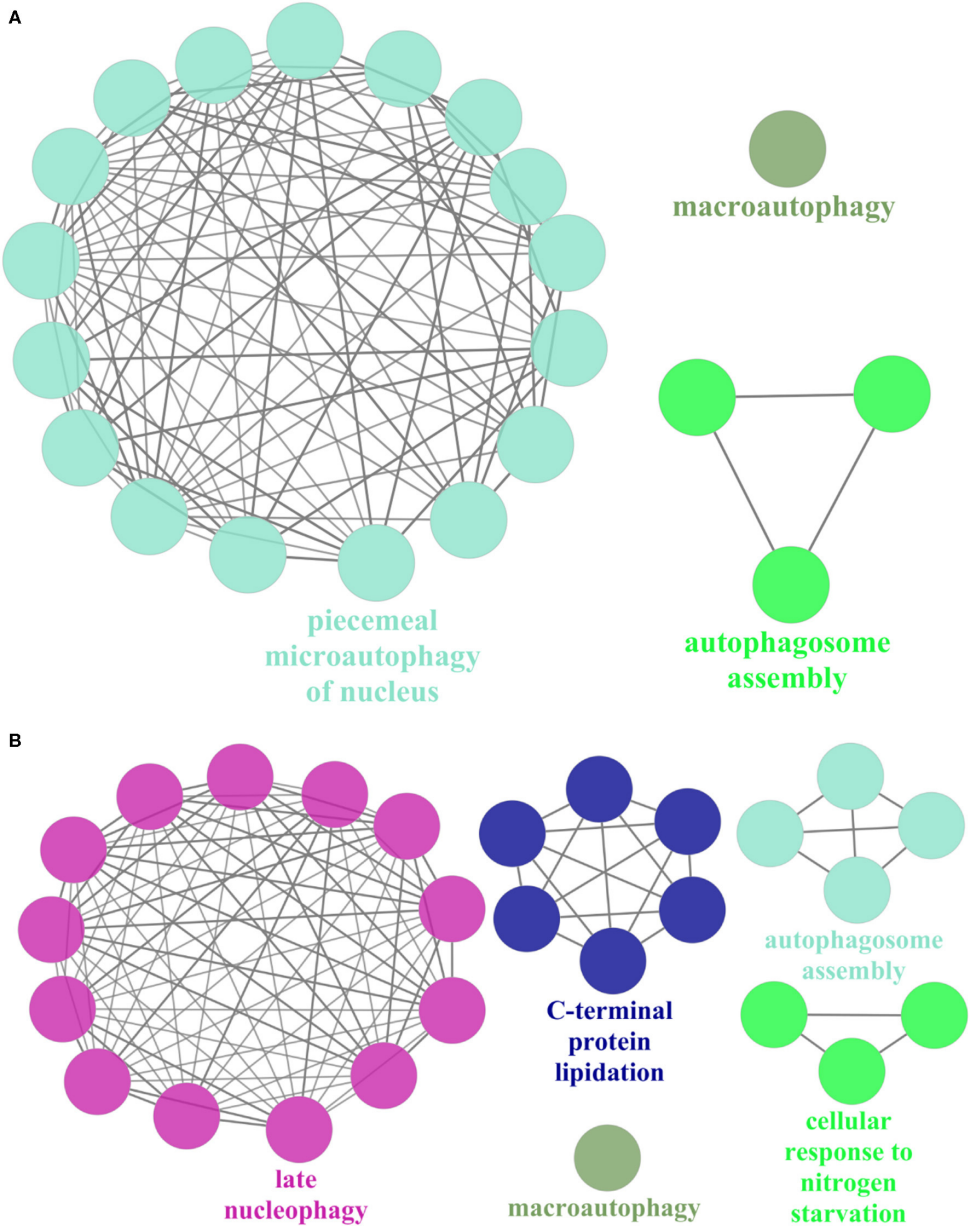
The gene enrichment of subnet in the *Saccharomyces cerevisiae* S288c. ClueGO is used to perform gene enrichment analysis on subnet **(A)** and expanded subnet **(B)** based on biological process (BP). The enrichment terms with statistical significance (*p* < 0.05) and the generated groups based on the relationships between terms are displayed separately. Each node represents a BP term, and each color represents a BP group. We remark the most significant BP term of each group in the figures. The edges indicate the relationships between the terms based on the similarity of their associated genes.

On the other hand, we randomly selected 1 of the 10 expanded subnets for gene enrichment analysis, the corresponding BP of which is autophagosome assembly. This subnet contains 7 nodes with 12 edges and 10 nodes with 29 edges after expansion, whose corresponding BP contains 74 proteins and 77 proteins after expansion. Refer to Figure 4A for the expanded subnet alignment, and there are three expanded protein pairs. We predicted that *S. cerevisiae* S288c proteins ATG8, ATG1, and ATG4 involved in the biological process autophagosome assembly, and this is indeed the case that the associated genes of this term in expanded subnet contain them (see Tables S3, S4). Based on the term group analysis in Figures 4B,C, we found that the term percentage of autophagosome assembly group increased but macroautophagy group reduced. From Figure 5, it can be seen that a new enrichment term organelle assembly appeared in the autophagosome assembly group; thereby, the term percentage of this group increased. This also shows that our expansion is practical. Besides, it also can be seen that the number of terms in the macroautophagy group is unchanged but the overall enriched terms increased, so the term percentage of this group reduced. Due to the addition of these three proteins, there are new groups such as late nucleophagy, C-terminal protein lipidation, and cellular response to nitrogen starvation that further illustrate that our subnet expansion algorithm is biologically significant.

## Discussion

In the study, we used BP as the standard for PPI network division, which made a preliminary restriction on the local alignment. It was helpful for finding more functional orthologous proteins and conserved functional modules. The BP associated PPI subnets were aligned by constructing pairwise similarity matrices of nodes. Considering that the network differences were mainly reflected in the node itself and the edge structure, we constructed the matrices based on the sequence similarity and graphlets similarity. In the related protein orthology matrix, we also added the matched edge information, in order to assess the sequence-edge similarity, and thus find more orthologous proteins and functional modules. By tuning the weight coefficients of the sequence-edge similarity and structure similarity, we found that the sequence-edge similarity made the major contribution to the local alignment. Moreover, the aligned proteins were annotated with the similar functions in the two species. In other words, NAIGO could achieve good alignments with biological significance by fully evaluating the orthology of the paired nodes and their 1-neighborhoods.

Based on the observations, we further expanded the BP-associated subnets with local alignments, and predicted the functions of newly aligned proteins accordingly. Because most human subnets were larger than the corresponding ones in the *S. cerevisiae* S288c, we tried to map the 1-neighborhoods of the *S. cerevisiae* subnets to the unaligned proteins in the human subnets. The result showed that such expansion was functionally effective. There were two probable reasons: on the one hand, the bigger human subnets provide references; on the other hand, we only expanded one layer. Similarly, we expanded the local alignment of the largest subnets to the global alignment, mapping the 1-neighborhoods in two species to each other until 1-neighborhoods of one species was empty. It offered the possibility to connect the fragmented subgraphs of a PPI network.

Taken together, the NAIGO algorithm could achieve local alignment by similarity matrix integrating the sequence-edge similarity and graphlets similarity and achieve global alignment by expanding the local alignment of the largest subnets. The PPI network alignments in the human and *S. cerevisiae* S288c showed that NAIGO outperformed some popular algorithms by aligning more orthologous proteins or more protein interactions. Although we focused on the alignment of two PPI networks in this study, NAIGO could also compare any other types of biological networks, such as gene regulatory networks, metabolic networks, and so on. Our algorithm is suitable for networks with thousands of nodes and edges without affecting the alignment results, and the scalability is still relatively strong.

On the other hand, NAIGO take the advantage of known prior knowledge for alignment, such as gene ontology and protein orthologous information, which provide biological references for the network alignment to a certain extent (Seah et al., 2014). However, if the alignment method relies on the biological information very much, it may miss some functional orthologs for the network pairs with similar sizes and topologies. On the other hand, if the method only relies on the topology information for the network pairs with large size differences, it will also result in the alignments with low functional quality. Thus, finding the optimal combination of biological information and topology information is still what we need to explore in the future.

## Data Availability Statement

The datasets generated for this study are available on request to the corresponding author.

## Author Contributions

JY and HZ conceived the project. LZ, JZ, and YZ implemented the experiments and analyzed the data. JL, JX, XB, NY, and GT prepared the data and performed literature search. LZ, JZ, and JY wrote the manuscript. All authors approved the final manuscript.

## Conflict of Interest

JL, NY, GT, and JY are currently employed by Geneis Beijing Co., Ltd. The remaining authors declare that the research was conducted in the absence of any commercial or financial relationships that could be construed as a potential conflict of interest.

## Supplementary Material

NAIGO is available at https://github.com/xujunlin123/nacode and Supplementary Material - NAIGO-SW.
