# Supplementary material for: NAIGO: An Improved Method to Align PPI Networks Based on Gene Ontology and Graphlets
Source: Front Bioeng Biotechnol. 2020 Jun 19;8:547. doi: 10.3389/fbioe.2020.00547 (PMC7318716; doi:10.3389/fbioe.2020.00547)
Supplement: Supplementary file 1 [file Table_1.DOCX]

Supplementary Material

# 1 Supplementary Figures and Tables

**1.1 Supplementary Figures**

## Not only did we find the conserved subnet pairs whose genes are all orthologs, but also found conserved subnets containing non- orthologs. We selected one of them for analysis: the proteins of Human subnet genes are RFC2, RFC5, RFC1, RFC4, RFC3, PCNA, POLE, and the proteins of Saccharomyces cerevisiae S288c subnet genes are RFC2, RFC3, RFC1, RFC4, RFC5, SMC6, SMC5, of which PCNA and SMC6, POLE and SMC5 are non-orthologous pairs (Figure S1). Through gene enrichment analyses of the two conserved subnets, we can know that the Human associated genes PCNA, POLE, RFC3, RFC4 and the Saccharomyces cerevisiae S288c associated genes RFC1, RFC2, RFC3, RFC4, RFC5 have the conserved function of' DNA strand elongation involved in DNA replication and DNA strand elongation (TABLE S2).


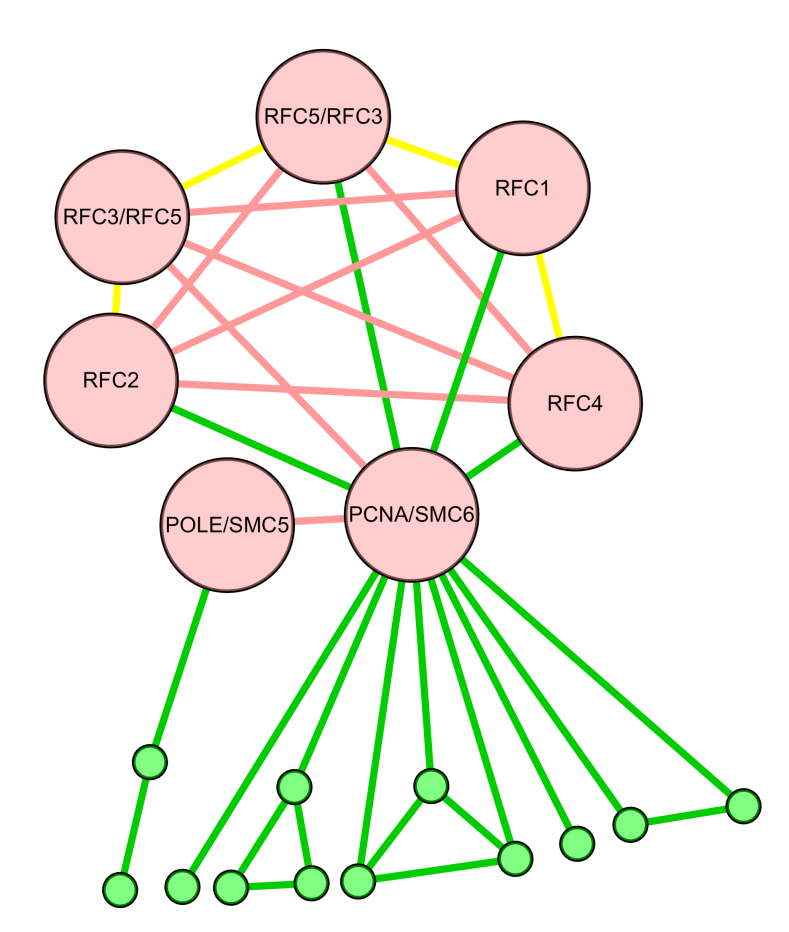


**Figure S1.** The conserved subnet. The yellow nodes and edges represent the proteins and interactions only existing in the Saccharomyces cerevisiae S288c, and the green nodes and edges represent the proteins and interactions only existing in the Human. The red nodes and edges form the conserved subnet of the two species. If the Human protein ‘A’ aligned to the Saccharomyces cerevisiae S288c protein ’B’ and ‘A’ is different from ‘B’, then we represent the red node as ‘A / B’. It should be noted that the proteins of the Saccharomyces cerevisiae S288c subnet are all conserved subnet proteins.

In addition, we display a larger conserved subnet between the two species (Figure S2).


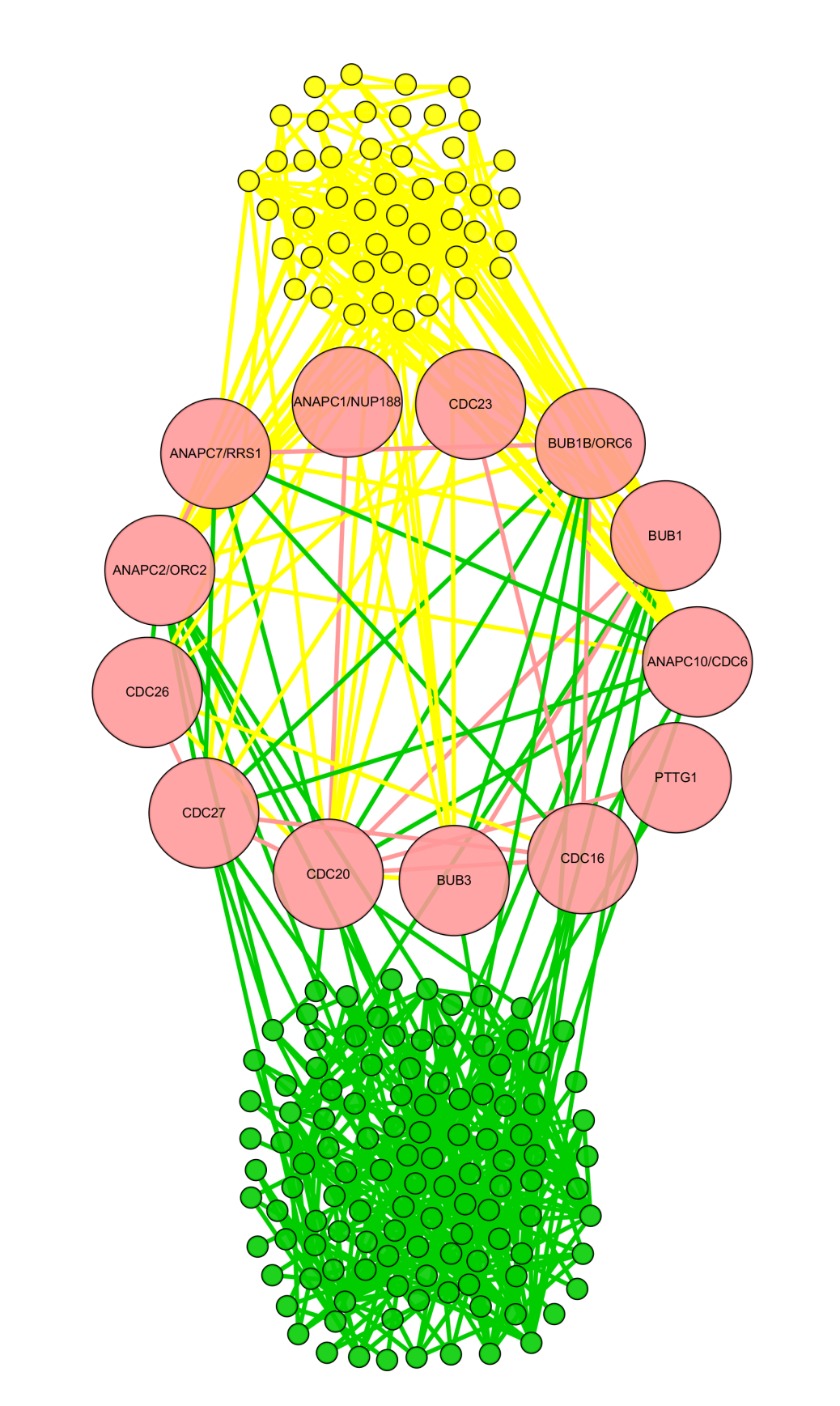


**Figure S2.** The conserved subnet. The yellow nodes and edges represent the proteins and interactions only existing in the Saccharomyces cerevisiae S288c, and the green nodes and edges represent the proteins and interactions only existing in the Human. The red nodes and edges form the conserved subnet of the two species. If the Human protein ‘A’ aligned to the Saccharomyces cerevisiae S288c protein ’B’ and ‘A’ is different from ‘B’, then we represent the red node as ‘A / B’.


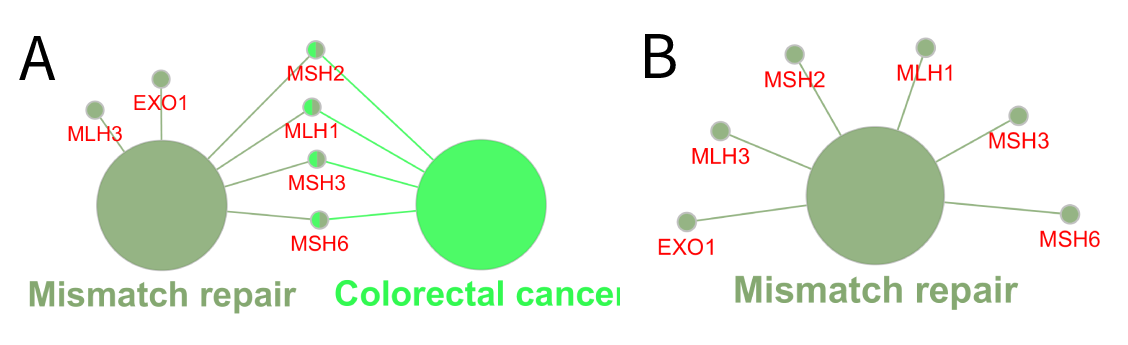


Figure S3. The gene enrichment of the Human (A) and Saccharomyces cerevisiae S288c (B) conserved subnet in Figure 2. ClueGO performs the gene enrichment analysis based on KEGG. Each colour represents an enrichment KEGG pathway (p-value < 0.05).


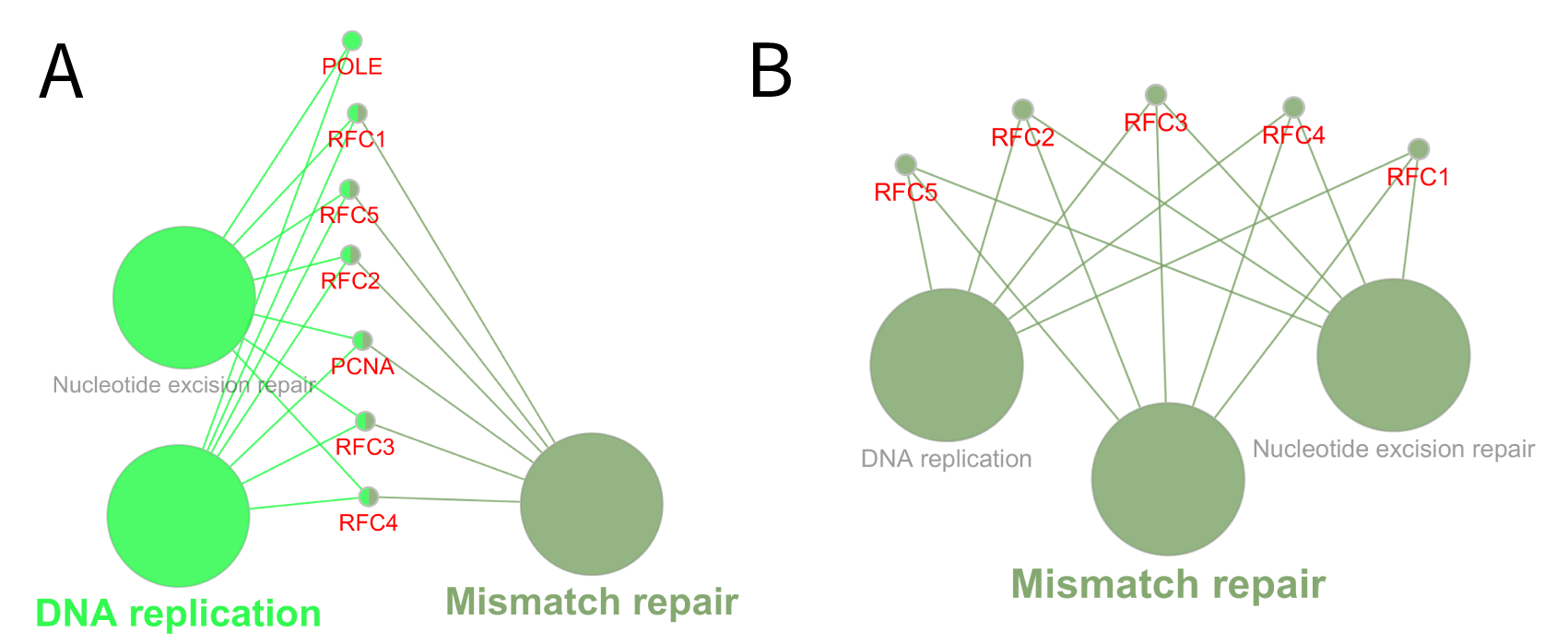


Figure S4. The gene enrichment of the Human (A) and Saccharomyces cerevisiae S288c (B) conserved subnet in Figure S1. ClueGO performs the gene enrichment analysis based on KEGG. Each colour represents an enrichment KEGG pathway (p-value < 0.05).


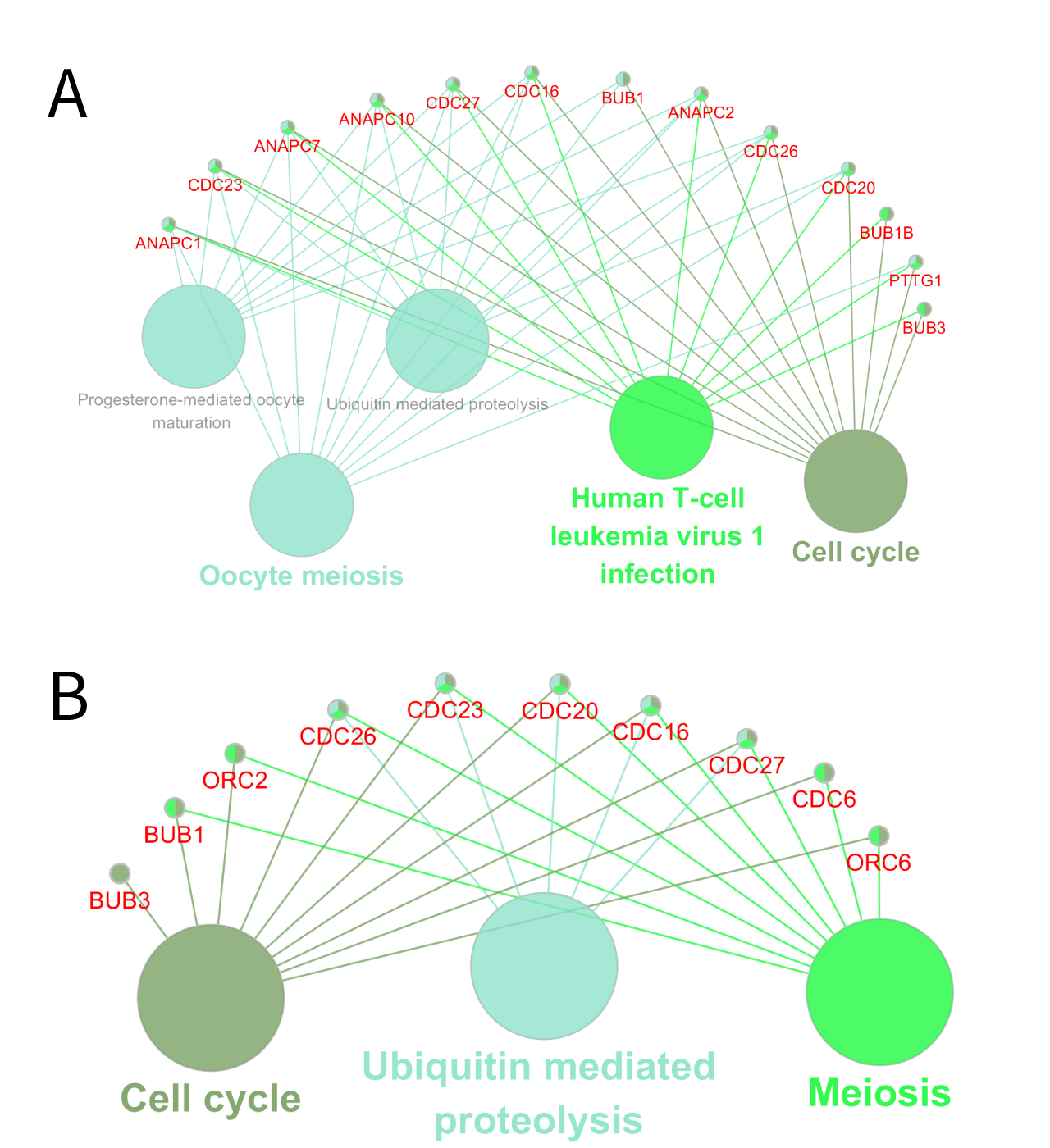


Figure S5. The gene enrichment of the Human (A) and Saccharomyces cerevisiae S288c (B) conserved subnet in Figure S2. ClueGO performs the gene enrichment analysis based on KEGG. Each colour represents an enrichment KEGG pathway (p-value < 0.05).

**1.2 Supplementary Tables**

**TABLE S1.** The defining parameters of 30 subnet pairs

| Subnet | Defined $\theta_{1}$ | Defined $\theta_{2}$ | Subnet | Defined $\theta_{1}$ | Defined $\theta_{2}$ | Subnet | Defined $\theta_{1}$ | Defined $\theta_{2}$ |
| --- | --- | --- | --- | --- | --- | --- | --- | --- |
| 1 | 0.8133333 | 0.186667 | 11 | 0.7313433 | 0.268657 | 21 | 0.9655172 | 0.034483 |
| 2 | 0.9298246 | 0.070175 | 12 | 0.9836066 | 0.016393 | 22 | 0.8768116 | 0.123188 |
| 3 | 0.9528302 | 0.04717 | 13 | 0.7931034 | 0.206897 | 23 | 0.8962963 | 0.103704 |
| 4 | 0.8571429 | 0.142857 | 14 | 0.8404255 | 0.159575 | 24 | 0.9363636 | 0.063636 |
| 5 | 0.9191176 | 0.080882 | 15 | 0.4889991 | 0.511001 | 25 | 0.7912088 | 0.208791 |
| 6 | 0.8807339 | 0.119266 | 16 | 0.8571429 | 0.142857 | 26 | 0.7923077 | 0.207692 |
| 7 | 0.4889991 | 0.511001 | 17 | 0.4889991 | 0.511001 | 27 | 0.9298246 | 0.070175 |
| 8 | 0.4889991 | 0.511001 | 18 | 0.8957055 | 0.104295 | 28 | 0.7068966 | 0.293103 |
| 9 | 0.858209 | 0.141791 | 19 | 0.7886179 | 0.211382 | 29 | 0.9528302 | 0.04717 |
| 10 | 0.8134328 | 0.186567 | 20 | 0.8707483 | 0.129252 | 30 | 0.9528302 | 0.04717 |

**TABLE S2.** The common GO terms and its associated Genes

| **Term name** | **Human associated Genes** | **Saccharomyces cerevisiae S288c associated Genes** |
| --- | --- | --- |
| DNA strand elongation involved in DNA replication | PCNA, POLE, RFC3, RFC4 | RFC1, RFC2, RFC3, RFC4, RFC5 |
| DNA strand elongation | PCNA, POLE, RFC3, RFC4 | RFC1, RFC2, RFC3, RFC4, RFC5 |

**TABLE S3.** The term group analysis of subnet.

| Group | GO Term | Term percentage | Associated genes |
| --- | --- | --- | --- |
| Group 1 | macroautophagy | 4.76% | ATG12, TG13, ATG14, ATG3, ATG5, ATG7, |
| Group 2 | autophagosome assembly  autophagosome organization  vacuole organization | 14.29% | ATG12, TG13, ATG5, ATG3, |
| Group 3 | piecemeal microautophagy of nucleus  mitophagy  protein lipidation  C-terminal protein lipidation  protein targeting to vacuole  lysosomal microautophagy  C-terminal protein amino acid modification  CVT pathway  lipoprotein metabolic process  lipoprotein biosynthetic process  post-translational protein modification  nucleophagy  late nucleophagy  mitochondrion disassembly  protein localization to vacuole  establishment of protein localization to vacuole  organelle disassembly | 80.95% | ATG12, TG13, ATG14, ATG3,  ATG5, ATG7 |

**TABLE S4.** The term group analysis of expanded subnet.

| Group | GO Term | Term percentage | Associated genes |
| --- | --- | --- | --- |
| Group 1 | macroautophagy | 3.70% | ATG1, TG12,ATG13, TG14, ATG3, ATG4, ATG5, ATG7, ATG8, |
|  |  |  |  |
| Group 2 | autophagosome assembly  organelle assembly  autophagosome organization  vacuole organization | 14.81% | ATG1, ATG12, ATG13, ATG3, ATG4, ATG5, ATG8, |
| Group 3 | cellular response to nitrogen starvation  cellular response to starvation  cellular response to nitrogen levels | 11.11% | ATG5, ATG7, ATG8 |
| Group 4 | C-terminal protein lipidation  C-terminal protein amino acid modification  lipoprotein metabolic process  lipoprotein biosynthetic process  post-translational protein modification  protein lipidation | 22.22% | ATG12, ATG3,  ATG4, ATG5, ATG7 |
| Group 5 | mitophagy  protein targeting to vacuole  vacuolar transport  lysosomal microautophagy  cellular component disassembly  CVT pathway  piecemeal microautophagy of nucleus  nucleophagy  late nucleophagy  mitochondrion disassembly  protein localization to vacuole  establishment of protein localization to vacuole  organelle disassembly | 48.15% | ATG1, ATG12, ATG13, TG14,  ATG3, ATG4, ATG5, ATG7, ATG8 |
